# Bluetongue Disease Control in Northern Ireland During 2017 and 2018

**DOI:** 10.3389/fvets.2019.00456

**Published:** 2019-12-17

**Authors:** Anastasia Georgaki, Archie Murchie, Ignatius McKeown, David Mercer, Sarah Millington, William Thurston, Karen Burns, Ben Cunningham, Valerie Harkin, Fraser Menzies

**Affiliations:** ^1^Veterinary Epidemiology Unit, Department of Agriculture Environment and Rural Affairs, Belfast, United Kingdom; ^2^Sustainable Agri-Food Sciences Division, Agri-Food and Biosciences Institute, Belfast, United Kingdom; ^3^Trade, Epizootics and Official Controls Division, Department of Agriculture Environment and Rural Affairs, Belfast, United Kingdom; ^4^Newtownards Divisional Veterinary Office, Department of Agriculture Environment and Rural Affairs, Belfast, United Kingdom; ^5^Atmospheric Dispersion and Air Quality, Met Office, Exeter, United Kingdom; ^6^Veterinary Sciences Division, Department of Virology, Agri-Food and Biosciences Institute, Belfast, United Kingdom

**Keywords:** risk assessment, surveillance, bluetongue, Northern Ireland, wind-borne, midges, *Culicoides*

## Abstract

Since the emergence of bluetongue virus in central and northern Europe in 2006, Northern Ireland's (NI) surveillance programme has evolved to include the use of risk assessments and simulation models to monitor the risk of bluetongue incursion. Livestock production is of high economic importance to NI as it exports approximately 75% of its agricultural produce. Its surveillance programme is designed to enable effective mitigation measures to be identified to minimize disease risk, and to provide additional assurances to protect NI's export markets in the European Union (EU) and third countries. Active surveillance employs an atmospheric dispersion model to assess the likelihood of wind-borne midge transfer from Great Britain (GB) to NI and to identify high risk areas. In these areas, the number of cattle tested for bluetongue is proportionally increased. Targeted surveillance is directed to ruminants imported from restricted countries and regions at risk of bluetongue. Targeted surveillance on high risk imports assists in early detection of disease as, despite all controls and preventive measures, legally imported animals may still carry the virus. In November 2018, a bluetongue-positive heifer was imported into NI. A case specific risk assessment was commissioned to estimate the likelihood of spread of bluetongue as a result of this incursion. November is the tail end of the midges' active period and therefore there was considerable uncertainty pertaining to the survival of midges inside a cattle shed and the potential for incubation of the virus in the vectors. An evidenced-based approach was adopted where temperature and midge abundance was monitored in order to minimize uncertainty and give an accurate estimate of the likelihood of virus spread to other animals following the arrival of the positive heifer. The heifer was destroyed and the evidence indicated that the risk of successful completion of the extrinsic cycle within the local midge population was negligible. This paper describes NI's surveillance programme between January 2017 and December 2018 and the case of a positive imported animal into the country. The importance of effective surveillance in early detection of threats and the usefulness of risk assessments is highlighted through the case study.

## Introduction

Bluetongue (BT) is an economically important, vector-borne, viral disease of ruminants, which can lead to high levels of mortality and abortions (particularly in sheep). Incursion of any of the 27 serotypes can lead to international restrictions on live animal trade, as outlined by the European Community and World Organization for Animal Health (also known as OIE) regulations. NI agriculture is mainly based on beef and dairy production systems (approximately 1.6 million cattle) (1) with 75% of its agricultural produce being exported, and hence very vulnerable to any restriction on such trade.

Bluetongue virus (BTV) is predominantly spread by certain species of biting *Culicoides* midges which vary according to geographical distribution. Midges become infected by feeding on the blood of viraemic ruminants and transmit BTV through subsequent feeding, which is required for the successful production of their eggs. Virus development in midges and transmission of BTV to ruminants are unable to occur in ambient temperatures below approximately 12°C (2) Incursion into regions free from BTV can occur through movement of viraemic animals into the area or by carriage of infected midges by wind plumes, both of which can occur over relatively long distances (3).

Historically, BTV had been mainly confined to the tropical and subtropical areas of the world. Within the last decade, there have been several outbreaks of BT, mainly of serotype 8, in central and northern Europe. After the 2008 BT virus serotype 8 (BTV8) epidemic which spread from France to Hungary and Sweden, there was a period without any cases being reported in central or northern Europe (2010–2014 inclusive). In August 2015 BTV8 re-emerged in France where it still remains present (as of April 2019). In 2018 BTV8 was detected in Switzerland and Germany and in March 2019 the virus was also found to be circulating in Belgium. At the end of March 2019, the whole of France, Switzerland and Belgium and a significant region of Western Germany were declared BT restricted zones according to Commission Regulation 1266/2007, meaning that formal regulations and restrictions on the movement of ruminants from such areas were applied.

The European Union introduced BT specific legislation in 2000 with Council Directive 2000/75, laying down provisions for the control and eradication of the disease. Subsequently Commission Regulation 1266/2007 was introduced, which outlined clear definitions of what constitutes a BT case and a BT outbreak. It establishes the minimum harmonized requirements for monitoring and surveillance of the disease in the European Community. This regulation clarifies that a case of BT is only confirmed if clinical signs or positive laboratory test results are the consequence of virus circulation in the holding in which the animal is kept. Member states are required to indicate circulation of the virus based on a set of epidemiological data.

This definition is not in complete accordance with the OIE terrestrial animal health code. The OIE code defines infection with BT as either the isolation of the virus from an animal or its products, or the detection of BTV antigen, RNA, or antibodies from an animal that shows clinical signs or is epidemiologically linked to a suspected or confirmed case.

EU Commission Regulation 1266/2007 was amended by Commission Implementing Regulation 456/2012 which changed the minimum requirements for monitoring and surveillance of BT. The current criteria for member states to demonstrate freedom from BT entails passive clinical surveillance and annual active surveillance including serological or virological testing of a representative sample of the bovine population, which is sufficient to detect disease prevalence of 20% with 95% confidence, within each 45 km by 45 km region. The legislation gives freedom to member states to formulate their own surveillance strategy within these criteria.

NI has remained free of BTV with the only detection of BT being in February 2008 when pregnant heifers imported from the Netherlands gave birth to calves that were seropositive and viraemic for BTV8. This was the first evidence of transplacental transmission of BTV8 from imported pregnant cows in NI. Surveillance on cattle and midges did not reveal any spread of the virus and only a single nulliparous *Culicoides* midge was caught on-farm during a nationwide survey in February 2008 (4).

The necessity to respond to legislative requirements and to address the risk of BT incursion into NI have been the drivers for developing a surveillance programme based on evidence and risk assessments.

### Surveillance

Since the BT epidemic in Europe in 2006 the risk of BT to NI has been monitored continuously. For 2017 and 2018 the threat of BT for NI came from wind-borne arrival of infected midges, or importation of infected ruminants. The closest infected country in 2017 and 2018 was France, for which wind-borne transfer of midges to NI was highly unlikely, but possible for GB. Risk assessments conducted by the Department for the Environment, Food and Rural Affairs (DEFRA, UK) in 2016 and 2017 estimated that the risk of introduction of BT to GB was low to medium, or, between 5% and 80% depending on the season of year (5).

Monitoring and surveillance programmes in NI are designed to mitigate these risks and consist of active surveillance of susceptible animals, targeted surveillance of imported animals and passive surveillance of reported suspect cases.

National vector surveillance was conducted in NI over 5 years (2008–2013), concluding after the end of the vector-free period in May 2013. A proportion of this dataset has been published (6).As the island of Ireland has always been BT free, and GB was declared BT free in 2011, an internal cost-benefit analysis concluded that sufficient information on vector species, distribution and seasonal profiles had been collected over the preceding 5 years to meet the requirements of Commission Regulation (EC) 1266/2007 with respect to vector monitoring outside of a restricted zone. Vector monitoring has thereafter been conducted on farm premises following a suspected BT case, in order to obtain specific localized information on vector presence and prevalence.

### Active Surveillance

April to November is the risk period during which temperature may be suitable for midge activity and virus replication according to studies conducted in NI (6). Model output showing the wind-borne spread of midges are provided by the Met Office daily from 1st April to 30th November using the Numerical Atmospheric dispersion Modeling Environment (NAME). NAME is a Lagrangian particle-trajectory model used to model the atmospheric transport and dispersion of a range of gases and particulates (7). In NAME, emissions into the atmosphere are simulated by creating a large number of computational particles where each computational particle represents, in this case, a certain number of midges. These particles are then advected along by the ambient three-dimensional wind field provided by the Met Office's Numerical Weather Prediction model with turbulent dispersion processes being simulated using random-walk methods. The computational particles can also evolve with time to account for various atmospheric processes that might affect midges in the atmosphere, including wash-out by precipitation.

NAME is run twice a day with model particles being released into the atmosphere over a 2 h period at sunrise and a 3 h period at sunset to represent the diel periodicity of midge activity (8) The particles are released from 10 m above ground level, a height assumed to be above the normal flight boundary layer of midges (8) The modeling takes into account the effects of temperature, wind speed and precipitation on midge activity at take-off, and the effect of precipitation on route on an hourly basis (9) In parallel to NAME runs for midges, NAME is run for the same source locations and time periods for tracer particles to indicate the movement of the air, not accounting for midge physiology and behavior. Three fixed locations in France, two fixed locations in GB, as well as locations in Belgium, Netherlands and Denmark, are used as source release sites for NAME. These locations are arbitrary and are used to illustrate the potential risk of incursion across the seas if BT was found near one of these coastal locations.

Active surveillance consists of a serological survey of a sample of susceptible cattle. Vaccination against BT is not permitted in NI except under license, therefore all homebred cattle are expected to be seronegative. As all high risk imported animals are tested, the most probable route of BTV infection for the endemic cattle population is through wind-borne incursion. Wind plumes carrying BTV infected midges could only arise from GB and hence the risk posed by spread from GB was monitored daily from two locations (Liverpool, England and Ayr, Scotland). This provided quantification of the risk posed if BTV became established in GB and also enabled annual monitoring for any undisclosed BTV incursion. The Normandy (France) dispersion point was monitored to evaluate possible spread to the Republic of Ireland as establishment there would pose a high risk of eventual spread to NI (results not presented). Monitoring this point also confirmed that wind-borne transfer of midges from Normandy to NI is highly unlikely.

**Figure 1:**
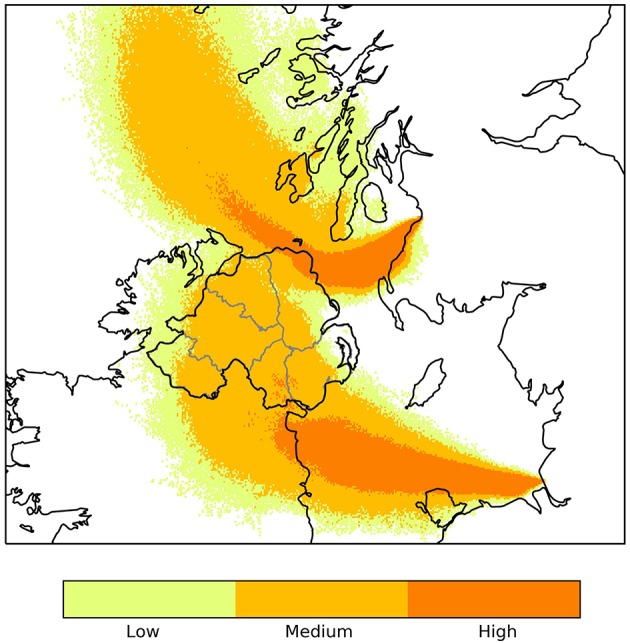
Output from the Numerical Atmospheric-dispersion Modeling Environment (NAME) showing relative concentration of midge particles released around sunset 29/06/2018 from hypothetical source locations in Ayr and Liverpool.

The Department of Agriculture Environment and Rural Affairs (DAERA) recorded high risk days for wind borne transfer of midges from GB. The risk of midges' arrival was considered high when the NAME model trajectory indicates possible transfer of midges from GB and/or France to the island of Ireland, and low when it indicates that tracer particles could arrive. When the model trajectory indicates possible transfer of midges over a NI county, in the morning, the evening, or both, this day is recorded as a high risk day for this county (Figure 1).

There were 22 high risk days for midge transfer from GB to NI in 2018 and 15 in 2017 (Table 1). The counties most at risk for midge transfer from the Liverpool monitoring point were Down and Antrim, while the counties most at risk for midges from Ayr were Antrim, Londonderry and Down. The model trajectory indicated possible dispersal of midges throughout all counties of NI on 4 occasions in 2018 and 3 in 2017. However, midges will attempt to land once over coastal areas and thus the NAME model output is only representative for wind-blown midge transport over water bodies. Overall, County Antrim is at higher risk from wind borne midges from GB followed by County Down and County Londonderry.

**Table 1:** Number of days where wind borne transfer of midges from GB to Northern Ireland was highly likely in 2017 and 2018.

	**Liverpool (England)**		**Ayr (Scotland)**	
**Year**	**High risk days**	**Low risk days**	**High risk days**	**Low risk days**
2017	4	5	11	9
2018	8	7	14	5

Sampling design was determined by the risk in each county, the cattle population, legislative requirements and historic surveillance recommendations. European Commission Regulation 1266/2007, which details the minimum BT surveillance requirements, states that the sample size must be able to detect a minimum prevalence of 20% with 95% confidence, within each 45 by 45 km region. NI's area is 14,129 km^2^ containing approximately 7 regions of 45 by 45 km within it (10) Historically, the OIE recommended detection of a minimum prevalence of 0.5% with 95% confidence, in the bovine population. NI's survey sample is based on this minimum prevalence within the total area of the country. The bovine population is structured in herds and this was selected as the most appropriate epidemiological unit for the survey of BT, a vector borne infectious disease. The sample size to detect 0.5% prevalence with 95% confidence, using an imperfect test with hypothetical sensitivity and specificity 95% and 100% respectively, was calculated to be 629 cattle herds (FREECALC2 from http://www.ausvet.com.au/). This sample size is able to detect a minimum prevalence of 3.5% with 95% confidence, within each 45 by 45 km region of NI. Modeling outputs suggested that testing four adult animals per herd would be optimal for detecting BT presence (11) To enable regional sampling to be systematic, a sample size proportionate to the total number of cattle present was drawn for each of the six counties, with an arbitrary 10% increase in sampling for the counties considered to be at higher risk from wind borne incursion (Table 2).

**Table 2:** NI's sample frame for BT serological surveillance.

**County**	**Divisional Veterinary Office (DVO)**	**Number of cattle herds**	**Number of cattle**	**Percentage of cattle**	**Adjusted percentage**	**Adjusted herd quota**
Armagh and Down	Armagh	2,427	157,175	10%	10%	60
Antrim	Ballymena	1,443	108,647	7%	8%	46
Antrim and Londonderry	Coleraine	2,673	204,227	13%	14%	86
Tyrone	Dungannon	2,910	175,314	11%	11%	67
Fermanagh	Enniskillen	3,082	153,824	10%	10%	59
Antrim	Mallusk	1,707	134,709	9%	9%	57
Londonderry	Londonderry	956	61,076	4%	4%	26
Down and Armagh	Newry	3,972	209,147	13%	15%	88
Down	Newtownards	2,023	165,250	11%	12%	70
Tyrone	Omagh	3,089	191,526	12%	12%	73
	Total	24,282	1,560,895	100%	106%	631

*DAERA has divided NI in 10 areas for administrative purposes. Administration of each division is provided by the Divisional Veterinary Office (DVO). The table shows the number of cattle herds by DVO, cattle population by DVO, percentage of total cattle population, percentage of cattle increased by 10% in high risk areas (adjusted percentage) and adjusted number of herds to be sampled for BT by DVO*.

Sampling took place in the vector-free period, mostly from January to March. This period was selected to coincide with the time of Brucellosis sampling on farms to improve cost efficiency of disease surveillance programmes and to satisfy the condition of detection of seroconversion. Cattle were tested for BT antibodies in serum by competitive Enzyme Linked Immunosorbent Assay (cELISA).

**Figure 2:**
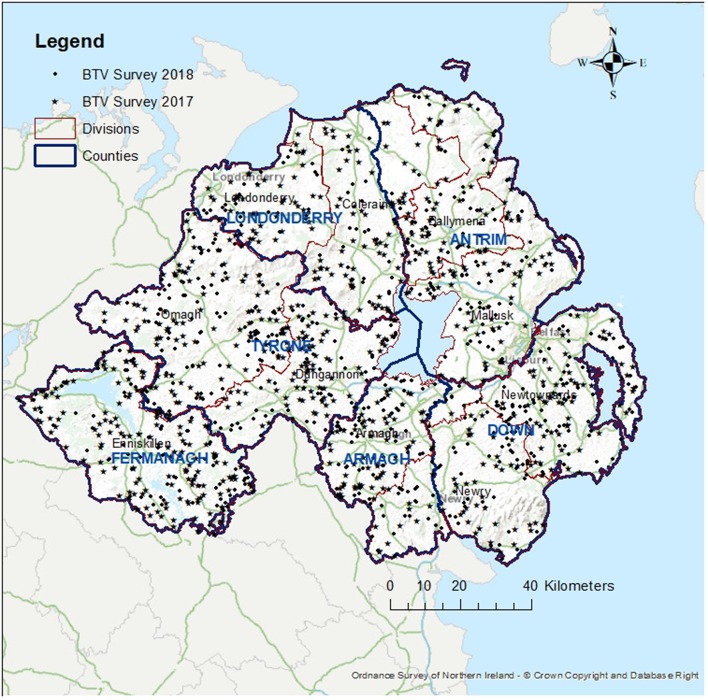
Map of Northern Ireland showing the location of the cattle farms sampled in 2017 (star) and in 2018 (dot), the six counties (blue boundaries) and the 10 administrative divisions of DAERA (red boundaries).

For this survey, 719 herds (2,876 animals) were tested for BT antibodies with cELISA in 2017 and 617 herds (2,468 animals) in 2018. Geographical distribution of the sample taken on years 2017 and 2018 is shown in Figure 2. One animal had a positive cELISA result in 2017 and one had a positive cELISA result in 2018. Both animals were tested again, in 13 and 28 days respectively, with cELISA, for presence of antibodies and, additionally, with reverse transcription polymerase chain reaction (RT-PCR) for presence of virus. At the re-tests the cELISA results were positive but the PCR results were negative for both animals. These results indicate the presence of antibodies against BT but absence of the virus. The 2017 animal was a cow born in GB in 2008 and imported to NI in 2013. As this was not an indigenous animal and its vaccination and exposure status outside NI could not be verified, it was excluded from the survey results. The 2018 animal was indigenous and had no apparent links to GB or other countries. This result triggered immediate movement restrictions on all the animals of the herd (25 cattle), compulsory housing, whole herd re-test and clinical and epidemiological investigations. When all actions were completed, there was no indication of either the presence or circulation of the virus. The animal showed no clinical signs and there was no evidence of a link with any other suspected or confirmed cases of BT in another country. Circulation of BTV was ruled out.

### Targeted Surveillance

Current DAERA policy is to test all ruminants imported from BT restricted countries and regions and countries at risk of BT in mainland Europe. In 2017 and 2018, cattle and sheep were imported from Austria, Belgium, Denmark, France, Germany, the Netherlands and Sweden. France reported BT cases in 2017 and 2018 and Germany in 2018. Imports from these countries were considered high risk.

Targeted surveillance consists of serological and virological testing of all susceptible species from at risk countries in mainland Europe within 5 to 7 days from arrival in NI. Animals arriving from BT restricted zones are tested immediately, either at the port of entry or at the farm of destination, and tested again 5 to 7 days later. Imported animals remain in isolation facilities until the results of the post-import tests are available. The tests conducted are RT-PCR on whole blood to detect viraemia and cELISA on serum to detect antibodies. The presence of antibodies in vaccinated animals provides reassurance of vaccine efficacy, while antibodies in unvaccinated animals may indicate natural infection.

In total, 236 consignments were identified for testing between January 2017 and December 2018. This resulted in 3,476 tests in consignments of cattle and sheep imported from mainland Europe and 41 tests in progeny of cows that were pregnant at the time of import. All except one (99.9%) of the RT-PCR tests were negative for BTV. The positive animal was a heifer imported from France in November 2018, and the case is outlined below. As expected, seroconversion was observed with cELISA in vaccinated animals from restricted zones where vaccination is a requirement for export. Antibodies were also detected in 10 other cattle at the post-import test while in isolation. Nine of them were imported from Austria between October 2017 and February 2018, and one from Denmark in August 2018. Austria had reported BTV4 outbreaks throughout 2016 and those animals were, most likely, previously immunized. These results were not surprising as BT antibodies can be detected at least 5 to 6 years after vaccination or natural infection (12, 13) In addition, out of 3,476 cELISA tests, two were initially positive but they were negative on re-test, which is explained by an estimated specificity of the cELISA test of 99.0% (95% CI 97.2–99.6%) (14).

### Case Study

In November 2018, following routine post import surveillance, a single heifer in a batch of 9 imported cattle from France tested positive for BTV using the OIE recommended RT-PCR Hofmann assay (15).

The animals were imported from France on 29th November, at a time when the whole country was a restricted zone and vaccination for BTV4 and BTV8 was compulsory for all exports. The export health certificates accompanying the animals indicated that they were vaccinated. The positive maiden heifer was in a batch of 5 heifers with the same destination herd. A breeding bull and 3 other cattle were also part of this load and went to separate holdings. In line with NI's surveillance programme, the imported cattle were blood sampled at the farm of destination on arrival and they were kept in an isolation facility on the farm. The batch of 5 heifers was tested with the confirmatory RT-PCR Shaw and Toussaint assay 6 days later, and the results confirmed that one heifer was positive for BTV8 (16, 17). Following this result in line with agreed disease control strategies the animal was humanely destroyed. The remaining heifers were negative for BTV. All imported cattle were also tested for antibodies by cELISA and found seropositive which was expected given their vaccination history. A confirmatory third post-import test on the 4 remaining imported heifers was completed in another 6 days, with the same results. The affected heifer was brought into a beef breeding herd of 126 animals which were kept separate, put under movement restriction, and tested twice, at 5 and 11 weeks after the import.

A case-specific veterinary risk assessment was authorized by the Chief Veterinary Officer (CVO) immediately after detection of the positive import, to examine the risk of BTV infection spreading from the positive heifer to the rest of the cohort. Only one possible transmission pathway was identified, which was midges inside the isolation shed feeding on the infected animal and transmitting the virus to the rest of the isolated heifers. Although it was reported that the isolation shed was treated with cypermethrin insecticide before the arrival of the imports, there was uncertainty around the presence and abundance of midges inside the shed. The maximum environmental temperature recorded by the Met Office station for the area ranged from 4.5°C to 10.2°C between 29th of November and 4th December. There was also uncertainty regarding the temperature range inside the isolation shed.

Entomological surveillance conducted in NI between 2008 and 2013, in accordance with regulation 1266/2007, identified that the vector-free period was between December and April (6). Nonetheless, variations exist from year to year and there were instances where midges had been found to still be active mid-December (6). Replication of the virus in the midge is likely to cease at temperatures below 12°C.

Although environmental temperature was below this limit, the temperature inside the shed could be higher, therefore survival of infected midges and transmission capacity inside the cattle shed could not be excluded. If the temperature inside the shed was, and remained above 15°C, the extrinsic incubation period for virus replication was estimated at 20 days (2).

It was therefore recommended to monitor the shed's temperature and midges' activity. Local surveillance of cattle and sheep on neighboring farms was not recommended on this occasion, as the likelihood of virus replication in midges outside the shed was considered negligible due to the incident occurring at the very end of the normal NI vector active period.

Two mains-powered (240 v) Onderstepoort UV light traps were set up on the farm. The first trap was placed inside the shed above the quarantined animals at a height of 2.7 m and the second trap was mounted on the outside wall of the shed at a height of 1.3 m. Four Tinytag TGP-4500 (Gemini Data Loggers, Chichester, UK) temperature loggers were similarly placed inside (*n* = 2) and outside (*n* = 2) of the shed close to the traps until the beginning of March. Temperature was logged each hour. *Culicoides* trapping commenced on 10 December 2018, with two 24 h samples taken each week until 28 February 2019, when the restrictions on the farm were lifted. Any *Culicoides* captured were identified to group level (*Obsoletus, Pulicaris, Impunctatus*, “other”) using wing patterning following the key of Boorman (18). The parity of the females (nulliparous or parous) was assessed using abdomen pigmentation following Dyce (19).

**Figure 3:**
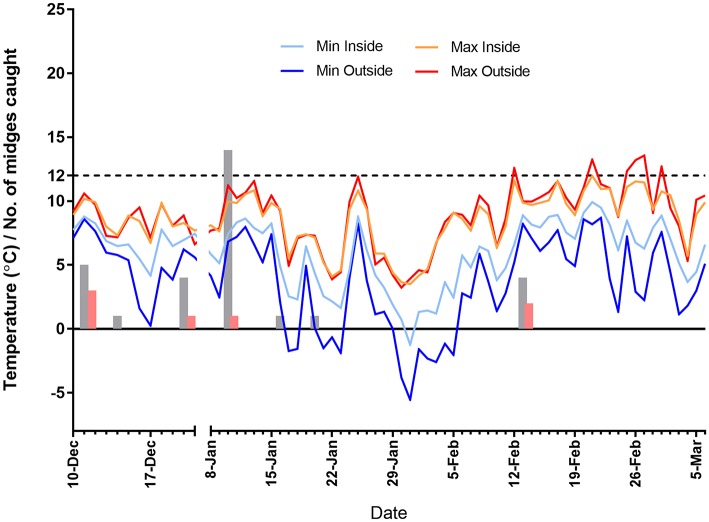
Average maximum and minimum hourly temperature recordings inside and outside the shed containing the quarantined animals, for the period of 10 December 2018 to 6 March January 2019 (no recordings were taken over the Christmas period). Light trap counts of female *Culicoides* are denoted by grey bars, with parous female counts denoted by red bars. The temperature limit to virus replication in midges is 12°C, which is denoted by a dashed line.

A total of 32 midges were caught in 43 trap collections. The majority of *Culicoides* collected (n = 30) were female Obsoletus group. Only a single male (Obsoletus group) and a single female “other” were collected. Most (*n* = 27) midges were trapped in the outside trap with only five Obsoletus females caught inside. A maximum of 14 Obsoletus group females were caught on 10 January 2019, with 11 in the outside trap and three on the inside one. A total of seven parous females were caught over the sampling period (all Obsoletus group) with a maximum of three caught in one 24-h period (Figure 3).

During the recording period the average temperature inside the shed was 7.2°C and outside was 6.3°C. Inside the shed the temperature ranged from −1.3°C to 12.0°C whilst outside this was −5.5°C to 13.6°C. The temperature was recorded above 12°C on the outside temperature loggers only, occurring on 6 days between mid-February and the beginning of March. For this particular shed, the effect of housing was not to increase upper temperatures but rather the body-heat of the livestock had the effect of moderating lower night-time temperatures (Figure 3).

Following negative results of all cattle tested on the farm both 5 and 11 weeks after the disposal of the positive heifer and the absence of a significant *Culicoides* midge population (i.e., reflecting Regulation 1266/2007: fewer than five parous females caught overnight in any one light trap collection during the surveillance period) the probability of BTV circulation was estimated to be negligible with a high degree of confidence.

## Discussion

Continuous and robust surveillance at, or above, legislative requirements and international standards is central to maintaining the BT free status of NI. Nevertheless, surveillance programmes need to be both efficient and cost effective. While the logistical approach utilized during BT surveillance in NI was very cost effective, the sample size utilized was higher than the minimum EU requirement. The additional sampling through use of the herd rather than the individual animal as the sampling unit was based partly on epidemiological rationale (as infection may be clustered within different herd management groups) and partly to provide additional assurance to non-EU countries thus protecting such export markets. This system builds on previous preliminary work in NI (20) and on experiences on risk based surveillance and midge based wind dispersion models documented elsewhere (21–23).

Improved efficiency can be achieved with a risk-based approach. In the case of serological survey for active surveillance, use of the NAME model has greatly improved the capacity to identify areas at higher risk of midges arriving from GB, and to increase BT surveillance toward these areas without increasing the sample size, therefore keeping the cost at the same level. NAME also models wind-borne transfer of midges from Normandy in the north of France, the country closest to NI with BT restriction zones in 2017 and 2018. It is highly unlikely that midges are transferred by the wind directly from France to Northern Ireland. This was confirmed by the model as there were no risk days for wind-borne dispersal of midges from France to NI.

During design of the surveillance system consideration must be given to practicalities that may hinder its implementation. The sampling frame as presented in Table 2 indicates that the target number of herds to be tested in a year is 631. However, during implementation, the actual number of herds tested deviated slightly from this target (719 and 617 herds tested in 2017 and 2018, respectively). Factors such as farming activities, co-ordination and timely release of resources forced alterations to the survey at the phase of implementation. This is not unexpected and a safety margin, to mitigate the risk of insufficient surveillance to maintain freedom, is necessary. In the current NI surveillance, the target number for the survey is well above the legislative requirements which provides flexibility during implementation. The sampling frame presented by the on-farm blood sampling for brucellosis surveillance provided an ideal and cost-effective platform with which BTV serological surveillance could be combined, which was not available to many of the other EU countries. However, this is an area of concern for the future as NI completes 5 years of brucellosis free status in 2020, with the requirements for brucellosis serological sampling being reduced therefore other surveillance protocols will have to be devised.

The Republic of Ireland (ROI) has examined the possibility of using blood samples from cull cows collected at abattoirs for country wide surveillance in order to demonstrate freedom from BTV and potentially other bovine viral infections (24). The study has indicated that blood samples from cull cows which were collected under the ROI brucellosis surveillance programme can also be used for BT surveillance. There were sufficient cull cows tested to allow a sample that was geographically stratified to cover the whole country. It was representative of the cattle population and its density, and the size of the sample was sufficiently large to satisfy EU requirements for disease freedom. This approach makes surveillance programmes less costly as more than one disease is tested from each sample. Sampling at abattoirs instead of farms has the additional advantages of saving time and resources and reducing the stress of a farm visit to cattle and farmers.

Another advantage of the NI situation is being part of an island, which mitigates against vector borne spread of BT to some degree as well as having physical limited access points for entry of livestock to the island as a whole. This enables much easier quantification of these incursion routes compared to mainland European countries, particularly in relation to use of assessing the risk from wind plumes. Application of some of the measures used in NI may be limited in such countries.

In the case of a positive import, the veterinary risk assessment identified transmission pathways, narrowed down the population of cattle at risk of infection and pinpointed areas of uncertainty. Unnecessary restrictions and testing of neighboring farms were avoided. Close collaboration between risk assessors and risk managers was essential to formulate the next steps of the disease control strategy. In this case, more information was needed to give an estimate of the probability of virus circulation in midges inside the cattle shed. The case happened at the end of the vector-active period and the presence of parous midges and virus circulation could not be excluded based on environmental temperature alone.

Resources were focused on gathering epidemiological data on the shed temperature and the capacity of midges to replicate and transmit BTV. Elucidation of this area of uncertainty increased confidence in the risk estimate.

Another situation where epidemiological data for BT surveillance is valuable is in the event of BT cases being identified within 150 km of NI, which could be in GB, the Isle of Man or the ROI. In such circumstances, parts of NI would be found inside the surveillance zone. This would have an impact on movement of animals and national and international trade. European Council Directive 2000/75 makes provision for changes to the boundaries of the zone when a duly substantiated request is made by a Member State (25). Having a well-documented, long standing surveillance programme would be fundamental, if NI wanted to request alteration of such zones on geographical, meteorological and epidemiological grounds.

## Data Availability Statement

The datasets generated for this study are available on request to the corresponding author.

## Ethics Statement

All samples from animals were obtained within an official context relating to disease control and surveillance. All sampling procedures complied with national and European regulations.

## Author Contributions

AG wrote the first draft of the manuscript and organized the wind-borne transfer of midges database and performed sample size calculations. FM and AG contributed in conception of the manuscript. AM wrote a section of the manuscript and provided entomological data and analysis. DM took accountability of field operations of the case study. SM and WT contributed to the manuscript, provided NAME data, and took accountability for NAME model outputs. KB, VH, and BC took accountability for laboratory testing. FM, IM, DM, AM, and VH critically revised the manuscript. All authors read and approved the submitted version.

### Conflict of Interest

The authors declare that the research was conducted in the absence of any commercial or financial relationships that could be construed as a potential conflict of interest.
